# New records of *Lophoproctus coecus* Pocock, 1894 (Diplopoda, Polyxenida, Lophoproctidae) extend the range of the genus *Lophoproctus*

**DOI:** 10.3897/zookeys.510.8668

**Published:** 2015-06-30

**Authors:** Megan Short

**Affiliations:** 1 Deakin University, 221 Burwood Highway, Burwood, Melbourne, Australia

**Keywords:** Penicillata, millipedes, geographic distribution, Caucasus, Crimea, Italy

## Abstract

The geographic distribution of the genus *Lophoproctus* Pocock, 1894 has greatly expanded with new records of the species *Lophoproctus coecus* Pocock, 1894, together with the reassignment of a number of millipedes formerly identified as *Lophoproctus lucidus* (Chalande, 1888). *Lophoproctus coecus* was found to be the sole representative of the family Lophoproctidae in collections examined from Crimea and the Caucasian region. The species was also identified from Iran and Kyrgyzstan. *Lophoproctus* specimens collected in Italy by Verhoeff were reassigned as *Lophoproctus coecus* with the exception of one specimen of *Lophoproctus jeanneli* (Brölemann, 1910) from Capri. These data were combined with all available information from the literature to look at the pattern of distribution of the four species in the genus. The range of the genus *Lophoproctus* extends from Portugal to Central Asia. *Lophoproctus coecus* is widespread from Italy eastward, while the morphologically very similar species *Lophoproctus lucidus* is confined to France and northern Africa. The two species have a narrow overlap in the Alpes Maritimes region of France. *Lophoproctus jeanneli* has a scattered coastal distribution around the Mediterranean Sea. The troglobitic species *Lophoproctus pagesi* (Condé, 1982) has only been recorded from a cave on Majorca, Spain.

## Introduction

**Figure 1.**
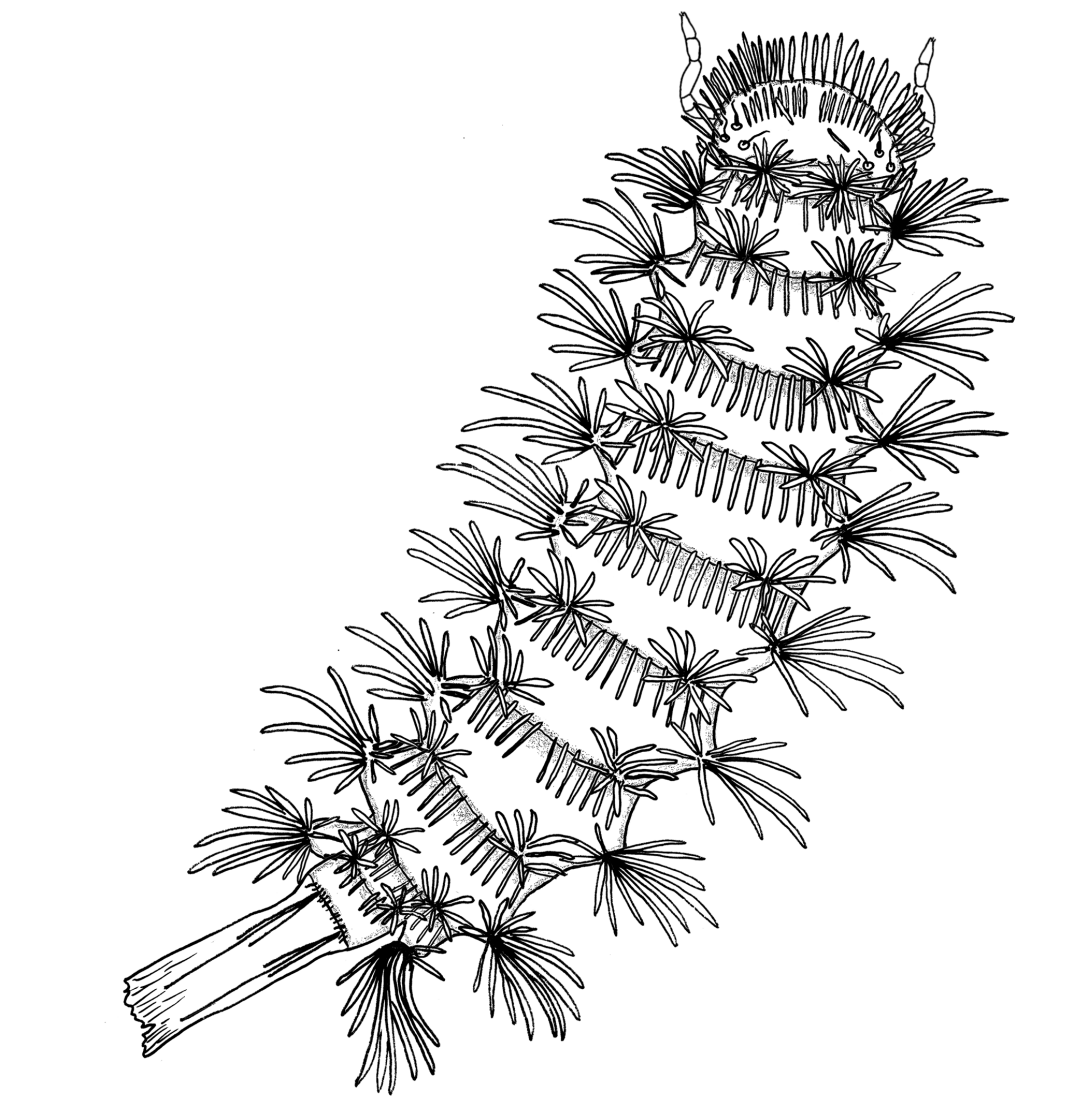
Habitus drawing of *Lophoproctus coecus* Pocock, 1894 showing typical morphology of the family Lophoproctidae.

Genera and species in the family Lophoproctidae Silvestri, 1897 have very similar morphology, with species adapted to an endogenous mode of life, being found in soil, caves and under stones. All Lophoproctidae lack ocelli, their integument lacks pigmentation and the 8^th^ antennal article is elongate. Species in the family also share the same arrangement of caudal trichomes and similar organisation of tergal trichomes (Fig. 1).

Characters used to determine genus and species of the family can be difficult to observe and include number and arrangement of antennal sensilla, number and arrangement of linguiform processes along the anterior margin of the labrum, structure of the telotarsus, leg setae, and tarsal spine. There are currently 5 genera: *Lophoproctus* Pocock, 1894, *Lophoturus* Brolemann, 1931, *Ancistroxenus* Schubart, 1947, *Lophoproctinus* Silvestri, 1948, and *Alloproctoides* Marquet & Condé, 1950. Two further genera *Barroxenus* Chamberlin, 1940 and *Trichoproctus* Silvestri, 1899, known only from single collections, are of uncertain status as they are inadequately described.

Pocock (1894) established the genus *Lophoproctus* for a species collected from soil at Nervi in Liguria, Italy. As the species lacked ocelli he called it *coecus* (‘blind’ in latin). Previously in 1888, Chalande had described the species Pollyxenus (sic) lucidus from Palalda, Eastern Pyrenees, France, which he initially described as having ocelli. In 1894 Silvestri identified *Polyxenus lucidus* from Italy, then later the same year recognising that the specimens had no ocelli he moved the species into the genus *Lophoproctus*. He further suggested that *Lophoproctus coecus* and *Lophoproctus lucidus* were synonymous (Silvestri 1894b) as did Verhoeff some years later (Verhoeff 1921). Both Silvestri and Verhoeff collected widely throughout Italy (Silvestri 1894a, 1894b, Verhoeff 1921, 1952) and identified all lophoproctids they found as *Lophoproctus lucidus* with the exception of those from Capri that Verhoeff mistakenly described as a new species *Lophoproctus litoralis* (Verhoeff, 1952). *Lophoproctus litoralis* was later determined to be *Lophoproctus jeanneli* (Brölemann, 1910) (Condé 1969). Condé (1978) re-examined material from Verhoeff’s collection from Isernia and Teramo, Zannone (Pontine Islands) and Sardinia, Italy, and noted that they differed from *Lophoproctus lucidus* in that they had a different arrangement of sensilla on antennal article VI which was also more elongate. On the basis of Condé’s description, Nguyen Duy-Jacquemin (1993) confirmed that these specimens were *Lophoproctus coecus*.

In the Caucasian and Crimean regions the previous records of *Lophoproctus* are by Lignau. He collected *Lophoproctus* in Krasnodar Polyanna (Lignau 1903), Crimea (Lignau 1905) and in Gagri (Lignau 1914) and although in his earlier papers he had identified the specimens incorrectly, in his 1911 paper he identified all as Polyxenus (Lophoproctus) lucidus (Lignau 1911). Subsequent species lists published all include *Lophoproctus lucidus* (Lohmander 1936, Kobakhidze 1965, Talikadze 1984) presumably based on Lignau’s early collections.

**Figure 2.**
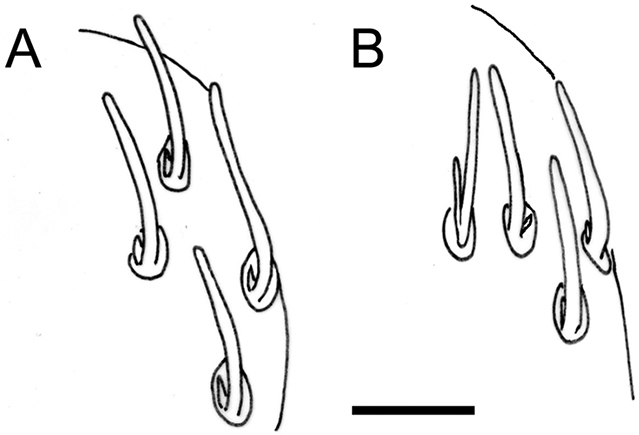
Typical pattern of basiconic sensilla on the right 6^th^ antennal article. **A** *Lophoproctus coecus* **B** *Lophoproctus lucidus*. The coeloconic sensilla are not visible due to angle of view. Scale bar: 20 µm (**A, B**).

In 1993, Nguyen Duy-Jacquemin redescribed *Lophoproctus coecus* from syntypes from Nervi, Italy that together with specimens collected in Zannone, Italy by Verhoeff and from Rome, Italy by Silvestri, confirmed that *Lophoproctus lucidus* and *Lophoproctus coecus* were not synonymous. Her paper clearly illustrates that the two species differ in arrangement of sensilla on antennal article VI (Fig. 2), structure of the median lobe on the anterior edge of the labrum, the number of ridges on the leg setae and the ratio of the length of the tarsal spine to length of the claw (Fig. 4). Initially described as a subspecies of *Lophoproctus lucidus*, *Lophoproctus jeanneli* is also found in the Mediterranean region of Europe but is easily distinguishable from other species of *Lophoproctus* by the presence of a denticle on the claw of the telotarsus.

**Figure 3.**
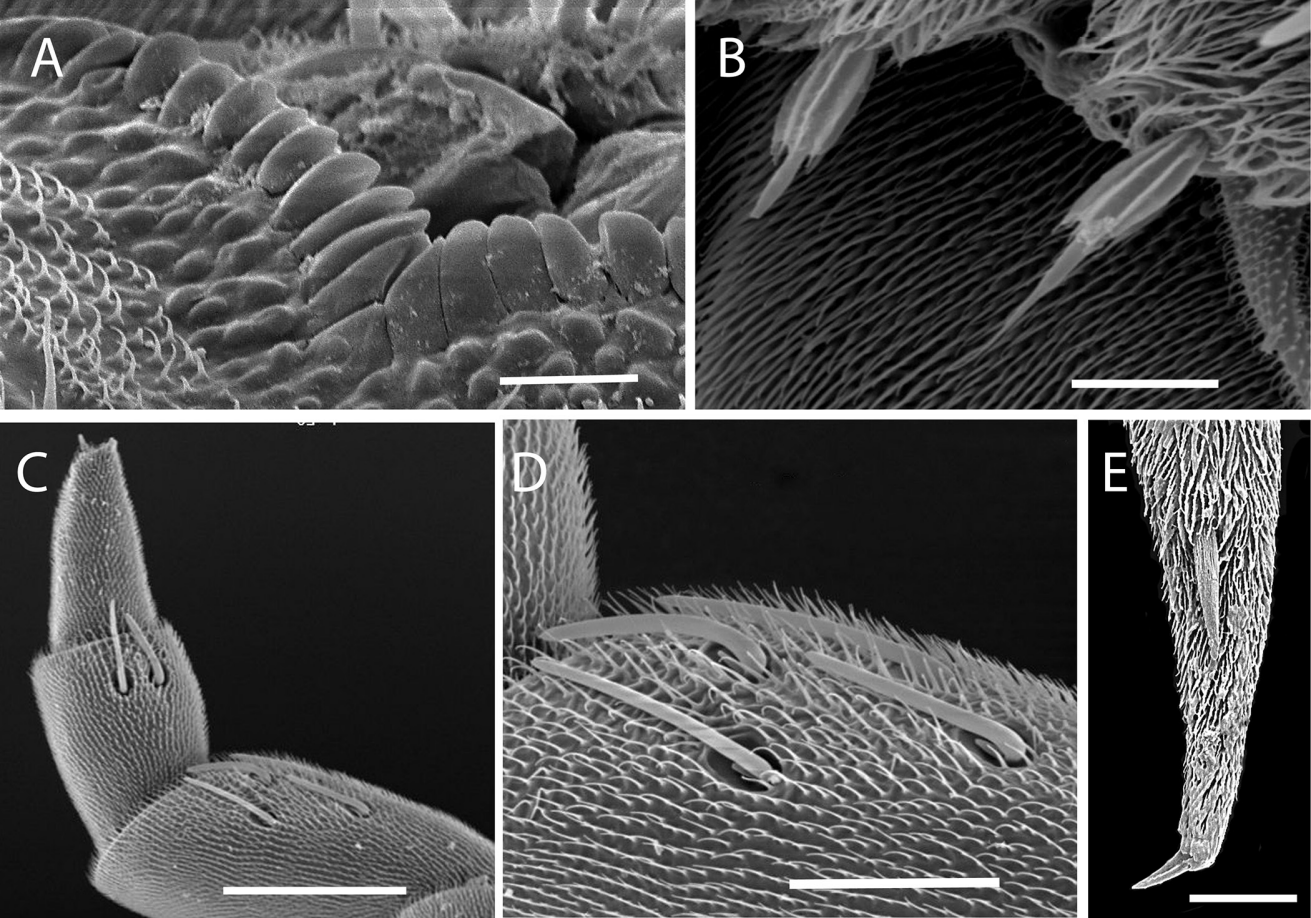
*Lophoproctus coecus* (Pocock, 1888), Krasnodar Province, Russia. Illustration of diagnostic features. **A** labrum showing triangular median linguiform process **B** typical setae on coxa **C** right antenna showing articles VI–VIII with sensilla **D** right antennal article VI showing arrangement of sensilla **E** tarsus 2 and telotarsus. Scale bars: 10 µm (**A, B**); 50 µm (**C**); 20 µm (**D, E**).

**Figure 4.**
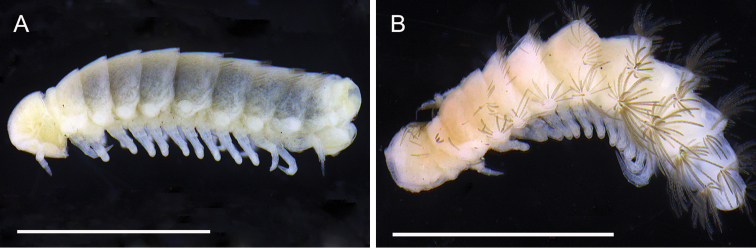
*Lophoproctus coecus* (Pocock, 1888) Krasnodar Province, Russia. **A** Lateral view showing antenna **B** Dorso-lateral view showing tergal trichomes. Scale bars: 1 mm.

The most recently described species *Lophoproctus pagesi* Condé, 1982, is a troglobitic species collected in caves on the island of Majorca, Spain. Specimens similar to *Lophoproctus pagesi* have also been collected from caves in Portugal. *Lophoproctus pagesi* differs from other species of *Lophoproctus* in details of the labrum, antennal sensilla and telotarsus. As well it has elongate antennae and legs typical of troglobitic species (Condé 1982, Nguyen Duy-Jacquemin 1993).

In this study I re-assessed specimens from The Zoological State Collection, Munich collected by Verhoeff in Italy and identified as *Lophoproctus lucidus*. I also identified Lophoproctidae from Crimea, Caucasus, Iran and Kyrgyzstan in the collection of the Zoological Museum of Moscow. These data were then combined with details obtained from the published literature on *Lophoproctus* species to determine distribution of species in the genus.

## Methods

The material examined for this study is lodged in the Zoological Museum of Moscow and the Zoological State Collection in Munich, Germany.

Specimens from the Zoological Museum of Moscow were examined and identified. All specimens were preserved in ethanol. These specimens were examined by light microscopy and scanning electron microscopy. For light microscopy, specimens were mounted on slides in Hoyer’s medium, dried at 60 °C and examined with an Olympus CX 41 compound microscope. Scanning electron micrographs were obtained of selected whole specimens that were dehydrated in a graded series of ethanol, 80%, 90% and 100%, then air-dried. Specimens were then mounted on stubs using adhesive tabs, sputter-coated with gold and examined with a Philips XL20 scanning electron microscope. Photographs of whole specimens were taken with a Leica Integrated Stereo-microscope System comprising a Leica 205C microscope with a DFC425 camera and 5000HDI dome illuminator. Images were stacked using Leica Application Suite and enhanced using Adobe Photoshop CS6.

Specimens from the Zoological State Collection, Munich, are slide mounts in Canada Balsam made by KW. Verhoeff. The slides lack both date of collection and site habitat details. The slides were examined by light microscopy using an Olympus CX41 compound microscope. Due to the thickness of the slide mounts, they could not be examined at magnifications higher than 400×.

As no coordinates were available for most of the material examined, Google Earth was used to provide an estimate of geographical position for mapping purposes (a table of localities with coordinates is available in supplementary material). A map of the distribution of all species in the genus *Lophoproctus* was generated using SimpleMappr (Shorthouse 2010). New records determined in this study were included together with all known published records. Many records in the literature, especially those by Verhoeff, Silvestri and Tabacaru are questionable and these have been treated separately.

## Results

Sixty collections of Polyxenida in the Zoological Museum of Moscow were examined and *Lophoproctus coecus* identified in 15. In most cases less than 5 specimens were collected at a site. No other species of Lophoproctidae were found.

Twenty slides from the Zoological State Collection, Munich (ZSM/Myr. 20031594–612, 615) all labelled as *Lophoproctus lucidus* were examined and 19 found to be *Lophoproctus coecus*. Slide ZSM/Myr. 20031615 contained a whole mount of *Lophoproctus jeanneli*.

**Figure 5.**
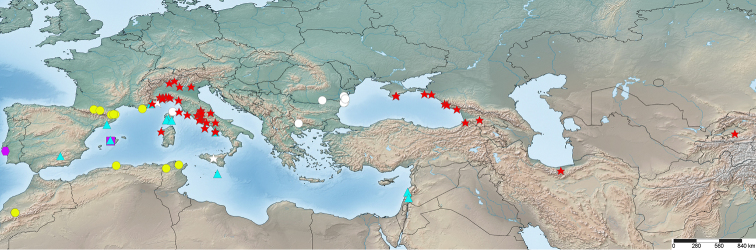
Map indicating geographic distribution of *Lophoproctus* species. Legend: red star = *Lophoproctus coecus*; yellow circle = *Lophoproctus lucidus*; white star = *Lophoproctus coecus*/*Lophoproctus lucidus*? (many in similar localities as *Lophoproctus coecus* and hence hidden); aqua triangle = *Lophoproctus jeanneli*; purple square = *Lophoproctus pagesi*; purple hexagon = Lophoproctus cf. pagesi; white circle = *Lophoproctus* sp. indet. Map created using SimpleMappr, http://www.simplemappr.net, (Shorthouse 2010).

The geographic distribution of all known localities of the genus *Lophoproctus* was plotted using the data listed below (Fig. 5).

## Systematics

### Order Polyxenida Lucas, 1840 Family Lophoproctidae Silvestri, 1897
*Lophoproctus* Pocock, 1894

#### 1. *Lophoproctus coecus* Pocock, 1894

**Records from literature.** Nervi, suburb of Genova, Italy, on the open hill-side behind the town, beneath stones, alt. 400–500 ft. (Pocock 1894); Grotta di San Antonino, Finale Ligure, Italy, leg. Ascenso 1950 (Nguyen Duy-Jacquemin 1993); Grotta di San Antonio =Antonino, Finale Ligure, Italy, leg. Comotti Baldan 13 Aug 1986 (Nguyen Duy-Jacquemin 1993); Grotta di Arma do Rian, Finalborgo, Italy, leg. Franciscolo 16 Mar 1952 (Nguyen Duy-Jacquemin 1993); Zannone, Ponziane Islands, Italy, leg. Condé 28–29 Jan 1966, 26–27 Feb 1966, 17 Jan 1967 (Nguyen Duy-Jacquemin 1993); Grotta di Nettuno, Porto Conte, Alghero, Sardinia, Italy, near the pier, 4 Oct 1955, leg. Condé (Nguyen Duy-Jacquemin 1993); Isernia, Italy, leg. Verhoeff (Condé 1982), ? same specimen as listed below; Teramo, Italy, leg. Verhoeff (Condé 1982), ? same specimen as listed below; Villa Pamphyli, Rome, Italy, leg. Silvestri Nov 1893 (Silvestri 1894a, reclassified Nguyen Duy-Jacquemin 1993).

**Unpublished record.** Nice (pers. comm. Nguyen Duy-Jacquemin 2014).

**Re-examined material collected in Italy by Verhoeff** (date of collection known for only 2 specimens). ZSM/Myr-20031594, Tivoli, Lazio; ZSM/Myr 20031595, Isernia, Molis; ZSM/Myr-20031596,Teramo, Abruzzo; ZSM/Myr-20031597, Portofino, Genova, Liguria; ZSM/Myr-20031598, Portofino, Genova, Liguria, molt; ZSM/Myr-20031599, Italy, molt; ZSM/Myr-20031600, Elba, Livorno, Toscana; ZSM/Myr-0031601, Chiesetal, Vestone, Brescia, Lombardia; ZSM/Myr-20031602, Isernia, Molise; ZSM/Myr-20031603, Ferrania, Liguria, 01.07.1933; ZSM/Myr-20031604, Mele, Genova, Liguria; ZSM/Myr-20031605, Monte Cimino, Soriano, Viterbo, Lazio; ZSM/Myr-20031606, Porto Santo Stefano, Grosseto, Toscana; ZSM/Myr-20031607, Veneto, Vicenza; ZSM/Myr-20031608, Ferrania, Liguria; ZSM/Myr-20031609, Santuario, Savona, Liguria; ZSM/Myr-20031610, Frigido, Toscana, from under stones at a mill ruin, April 1907; ZSM/Myr-20031611, illegible labelling; ZSM/Myr-20031612 Capri (No 27), Napoli, Campania.

**New material from Zoological Museum of Moscow.** Nikita Botanical Garden, Cape Martyan, near Yalta, Crimea, 4 Nov 1947, leg. M.S. Ghilarov; Gurzuf, Yalta, Crimea, Jun–Sep 1947, leg. M.S. Ghilarov (2 vials); Utrish Nature Reserve, Krasnodar province, Russia, oak hornbeam forest, 15 Jun 2013, leg. I. Tuf; Utrish Nature Reserve, Krasnodar province, Russia, hornbeam forest, 14 Jun 2013, leg. I. Tuf; Goryachy Klyuch, Mtn ridge, Markotkh plateau, Krasnodar province, Russia, 3 Jul 1956, leg. M.S. Ghilarov; Dagomys, Sochi, Krasnodar province, Russia, *Quercus* shrub, *Carpinus*, *Fagus* etc., 18 May 1983, leg. S. Golovatch; on road 2 km N of Dagomys, Krasnodar province, Russia, 2 Jun 2014, leg. M. Potapov; Cave “Our Lady”, ca 8 km from Khosta Sochi, Krasnodar province, Russia, *Buxus*, *Fagus*, *Acer* etc., forest near entrance, litter and under stones, 16 May 1985, leg. S. Golovatch; Khosta, Sochi region, Krasnodar province, Russia, *Sambucus*, 26 Jun 1956, leg. M.S. Ghilarov; Ris Forest, Bobcai east, Gumista River, Abkhazia, Russia, litter, 5 Jun 1982, leg. J. Bohàc; environs of Keda, Adjaria, Georgia, *Picea* and deciduous forest, 1 Oct 1975, A. Druk; Nedzura River valley 8km SE of Akhaldaba, Borzhomi district, Georgia, *Picea*, *Carpinus* and *Fagus* forest, litter, logs, 12 May 1983, leg. S. Golovatch; Arslanbob, Fergana mountain range, environs of Yarodar, Kyrgyzstan, dry limestone slopes with grass, under stones, 28 Sep 1983, leg. K. Eskov; Sari, Mazanderan province, Iran, *Quercus* and *Carpinus* forest, 11 Apr 2013, leg. M. Mehrafrooz.

**Distribution.** South-East France, Italy, Russia, Georgia, Iran and Kyrgyzstan.

#### 2. *Lophoproctus lucidus* (Chalande, 1888)

**Records from literature.** Palalda (now Amélie-les-Bains-Palalda), Pyrénées-Orientales, France, in soil under litter layer in oak woods, leg. Chalande (Chalande 1888); cave Gourgue,Canton Aspet, No. 229, Haute-Garonne, France, (Nguyen Duy-Jacquemin 1993); Baumo de las Fadas, Canton du Barjac, Dept du Gard, France, 26 Aug 1909, leg. Brölemann (Brölemann 1910); Albères, France, 1926, leg. Brolemann (Condé 1950); Banyuls sur Mer, France, leg. Brölemann (Nguyen Duy-Jacquemin 1993); Hyères, France leg, A Dollfus (Nguyen Duy-Jacquemin 1993); Ariège, France, leg. Nguyen Duy-Jacquemin (Nguyen Duy-Jacquemin 2000); Souk el Arba, Jendouba district, Tunisia, 30 Mar 1896, leg. Silvestri (Silvestri 1896); La Pérouse (now known as Tamentfoust), Dar El Beïda district of Algiers, Algeria, in wave washed and dry plant material (Seurat 1930); Marrakech, Morocco, 10 Dec 1950, (Condé 1954); Marrakech, Morocco, Jardin de l’ Aguedal, near the Mechouar (Condé 1954); Marrakech, Morocco, Jardin de la Bahia (Condé 1954); Marrakech, Morocco, Parc de la Villa Majorelle, under flower pots and stones, 10 Dec 1950 (Condé 1954).

**Unpublished records.** El Ghazalaf Ariana, nr. Tunis, Tunisia, garden of private house in earth, beneath *Cydonia* tree (pers. comm. N. Akkari, 2014).

**New material.** La Parc Phoenix, Nice, France, in cold greenhouse, Jan 2014, leg. JM Lemaire.

**Distribution.** France, Tunisia, Algeria, Morocco.

#### 3. *Lophoproctus jeanneli* (Brölemann, 1010)

**Re-examined material collected by Verhoeff** (date not given). ZSM/Myr-20031615: Corsica, France.

**Records from literature.** Baume (grotto) du Colombier, Alpes-Maritimes, commune de Roquefort-les-Pins, canton de Bar-sur-Loup, France, 17 Sep 1905, 27 Sep 1908 (Brölemann 1910); Grotte de la Chèvre d’Or, canton de Bar-sur-le-Loup, France 25 Nov 1987, leg. V. Aellen, (Condé 1989); City park, Barcelona, Spain, Sep 1950 leg. Condé (Condé 1954); Lower Gravona River, left bank of western arm of river, opposite Canapajolo, Corsica, France (Condé 1953); Pointe de Porticcio, sur la côte S. du golfe d’Ajaccio, near houses, 1 km to south west of Fallaccioli, Corsica, France (Condé 1953); Togna, commune de Sari-di-Porto Vecchio, at the edge of a ravine and a garden well, Corsica, France, (Condé 1953); Cueva de la Moriguilla (Vilacarrilo), Andalucia, Spain (Golovatch and Mauries 2013); Esporlas near Palma, Majorca, Balaeric Islands, Spain, at irrigation canal overgrown with dry compact rootlets 17 Aug–23 Sept 1954, leg. J. Pagès (Condé 1955); near Bagno di Tiberio, beach, Capri, Italy, leg. Verhoeff (Condé 1969); Malta leg. Silvestri (Nguyen Duy-Jacquemin 1993, with reservation); near Dékouané, 7 Km to east of Beirut, Lebanon April 1952, leg. PJ. Corset, (Condé 1954, Nguyen Duy-Jacquemin 1993 with reservation); Tel Dan, Israel, 26 Dec 1963, leg. G. Levy (Condé and Nguyen Duy-Jacquemin 1970).

**Distribution.** France (mainland and Corsica), Spain (mainland and Majorca), Italy (Capri), Malta, Lebanon, Israel.

#### 4. *Lophoproctus lucidus*/*coecus*?

Specimens identified as *Lophoproctus lucidus* but likely to be *Lophoproctus coecus* as Silvestri and Verhoeff thought the two species were synonymous.

**Records from literature.** Bevagna, Umbria, Italy, in meadow and forest, late October 1893, leg. Silvestri (Silvestri 1894a); Woods at Madama and Acquacetosa, near Rome, Italy, on ground in plant debris, in forest and open places, leg. Silvestri, November 1893 (Silvestri 1894a); at Colle Pezzo, Mt. Martano, Umbria, Italy 15 Oct 1893, leg. Silvestri (Silvestri 1894a); Medol Casello, Lombardy, Italy, on floor of cave 1935 –1940, leg. various unnamed (Manfredi 1940): Sicily, Italy, leg. Silvestri (Silvestri 1898); Mt. Schignano, Italy, in soil among plant debris, not only in forests, but also in open places, 15 Oct 1893, leg. Silvestri (Silvestri 1894a); Syracuse, Sicily, Italy, 1962–1968, leg. Institute of Zoology of Catania (Strasser 1970); Ciminà, Aspromonte, Calabria, Italy 25 Oct 1966, leg. G. Osella (Strasser 1970); St Remo, Italy under stones in an olive terrace 7–21 April 1907, leg. Verhoeff (Verhoeff 1921); Pegli, Italy in creek valley 7–21 April 1907, leg. Verhoeff (Verhoeff 1921); Massa, Carrara, Italy, in sandstone gorge 7–21 April 1907, leg. Verhoeff (Verhoeff 1921); St. Margherita, Italy, in chestnut wood 7–21 April 1907, leg. Verhoeff (Verhoeff 1921).

#### 5. *Lophoproctus* sp. indet., reported as *Lophoproctus lucidus*

**Records from literature never formally identified** (pers. comm. Nguyen Duy-Jacquemin, 2014): Pădurea Comorova, Romania (Tabacaru 1966); Mangalia, Romania, 20 Nov 1963 (Tabacaru 1966); Pădurea Hagieni, Romania, 17 May 1963, (Tabacaru 1966); Canaraua de pe Graniţă - comuna Băneasa, Romania 2 Aug 1962, leg. Dumitrescu et al. (Tabacaru 1966); Cave Gura Dobrogii litoclazic, Romania 17 Jun 1963 leg. Dumitrescu et al. (Tabacaru 1966); Cave Gura Dobrogii, Romania, at entrance 17 Sep 1963 leg. Dumitrescu et al. (Tabacaru 1966); Casian, Romania 27 Jul 1962, 7 May 1963, 30 Aug 1964 leg. Dumitrescu et al. (Tabacaru 1966).

**Further record from literature, identification uncertain.** Marine de Sisco, Corsica, France, 3 Sept 1942, leg. P. Remy (Verhoeff 1943).

#### 6. *Lophoproctus* sp. indet.

**Record from literature.** Kalimantsi, South Pirin Mountains,Bulgaria, in ant nests 1 Mar 2003, leg. Lapeva-Gjonova (Stoev and Lapeva-Gjonova 2006)

#### 7. *Lophoproctus pagesi* (Condé, 1982)

**Records from literature.** Cueva de Genova near to Palma, Majorca, Balearic Islands, Spain (Condé 1982); Cueva de Bellver, Palma, Majorca, Balearic Islands, Spain, unpublished, Condé det. (Nguyen Duy-Jacquemin 1993);

Lophoproctus cf. pagesi : “Gruta do Fumo”, Parque Natural da Arrábida, Portugal (Cardoso et al. 2008, Nguyen Duy-Jacquemin 2014).

**Distribution.** Caves on Majorca (Lophoproctus cf. pagesi – cave in Portugal).

## Discussion

*Lophoproctus coecus* has previously been considered to occupy a scattered range within the Central Mediterranean region, but the results of this study indicate that the species is widespread throughout Europe particularly in Eastern Europe with its distribution extending into Central Asia. *Lophoproctus lucidus* in comparison seems limited to the Southern France, as well as Morocco, Algeria and Tunesia in Northern Africa. The identification of *Lophoproctus jeanneli* from Capri, Italy reinforces the Mediterranean coastal distribution previously noted by Kime and Enghoff (2011). It is of interest that species within the genus may overlap in their geographic distribution with both *Lophoproctus jeanneli* and *Lophoproctus coecus* being found in Capri and in the Alpes Maritimes region of France. *Lophoproctus pagesi* and *Lophoproctus jeanneli* both occur on the island of Majorca, with *Lophoproctus pagesi* restricted to caves while *Lophoproctus jeanneli* was found in humid, sunny hilly areas.

A number of identifications were unable to be checked. In the case of the *Lophoproctus* identified by Lignau as *Lophoproctus lucidus*, specimens identified as *Lophoproctus coecus* in this study were found at all 3 of Lignau’s collection areas, indicating that it is most probable that the specimens collected by Lignau were in fact *Lophoproctus coecus*. Prior to publication of Nguyen Duy-Jacquemin (1993), the difference between *Lophoproctus coecus* and *Lophoproctus lucidus* was not understood with Silvestri (1894b) considering the two species synonymous. Hence, until it can be confirmed, the identification of specimens of *Lophoproctus* as *Lophoproctus lucidus*, from Romania (Tabacaru 1966) and Sicily (Silvestri 1903, Strasser 1970) must remain questionable. *Lophoproctus* has also been collected from Bulgaria but has not yet been identified to species (Stoev and Lapeva-Gjonova 2006).

**Figure 6.**
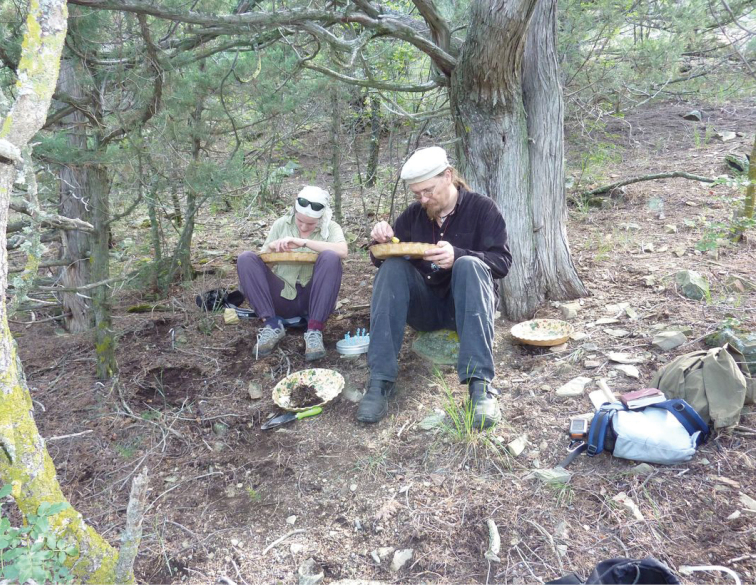
Ivan H. Tuf and Daria Kuznetsova collecting invertebrates including *Lophoproctus coecus* from sieved soil and litter, Utrish Nature Reserve, Krasnodar province, Russia.

The distribution map (Fig. 5) indicates very clearly that there is a big gap in our knowledge of *Lophoproctus* in Greece, the Balkans and Turkey. It is predicted that *Lophoproctus coecus* does occur in these three locations, and that *Lophoproctus jeanneli* may also occur in coastal regions. All species except the troglobitic *Lophoproctus pagesi* have the ability to live in many of the habitats in these areas of Europe and Asia. Unfortunately there is limited information available to guide collection of these tiny millipedes. In most cases millipedes in this study were collected by hand collecting in the field, or by sieving of litter and/or soil followed by direct collection from a tray of sieved material (Fig. 6). Recently in Dagomys, Russia, *Lophoproctus coecus* was collected from forest litter by funnel extraction (M. Potapov pers comm. 2014). Habitats from which *Lophoproctus* has been collected vary from maquis and forest litter, top layer of soil, cave floors, to under stones and logs, and in ants nests (Stoev and Lapeva-Gjonova 2006). In the case of *Lophoproctus coecus* from Kyrgyzstan, the single specimen was collected from under stones on a dry grassy limestone slope, a similar habitat to that of the type collected by Pocock (1894), ‘open hillside beneath stones’. As well as the above mentioned habitats, *Lophoproctus lucidus* has been found in North African cities in city parks, in gardens and under pot plants and stones (N Akkari pers comm. 2014, Condé 1954), and near Algiers on the beach in accretions of both dry and damp marine plant material (Condé 1954). *Lophoproctus jeanneli* has been collected in abundance from cracks in bricks and under dry stones on the ground in heavy shade in the Barcelona City Park (Condé 1954) as well as on the sea shore (Verhoeff 1952).

Methods of dispersal have not been studied, but it is probable that the presence of *Lophoproctus lucidus* in North African cities is due to anthropogenic activities dating back to the French colonial period as millipedes confirmed to be *Lophoproctus lucidus* appear limited to Southern France and centres of French colonial activity in North Africa. Polyxenida are thought to passively disperse via the wind and incidental attachment to the feathers of birds. It is likely in the case of soil and litter dwelling lophoproctids that dispersal via wind is less common as they do not appear to be living in elevated situations in trees and bushes in contrast to Polyxenida from the families Synxenidae and Polyxenidae. However, limited methods of dispersal do not seem to have restricted the geographic ranges of species of the genus *Lophoproctus*, especially *Lophoproctus coecus*.

## Supplementary materials

Supplementary material 1 Record details of all *Lophoproctus* collections. Data type: Comma-separated values files (CSV) Explanation note: A record of all specimens of *Lophoproctus* examined, or mentioned in the literature, is available. This dataset is made available under the Open Database License (http://opendatacommons.org/licenses/odbl/1.0/). The Open Database License (ODbL) is a license agreement intended to allow users to freely share, modify, and use this Dataset while maintaining this same freedom for others, provided that the original source and author(s) are credited.Author: Megan Short
